# Identification of Adeno-Associate Virus (AAV) Serotype for Endometriosis Therapy and Effect of AAV-Mediated RNAi Delivery on Gene Expression and Cell Proliferation in In Vitro Endometrial Cell Culture

**DOI:** 10.3390/microorganisms13092144

**Published:** 2025-09-13

**Authors:** Jin Kyung Baek, Jaekyung Lee, Yun Soo Chung, Seokkyo Seo

**Affiliations:** 1 Department of Obstetrics and Gynecology, Severance Hospital, Yonsei University College of Medicine, 50-1 Yonsei-ro, Seodaemun-gu, Seoul 03722, Republic of Korea; 2 Department of Medicine, Yonsei University College of Medicine, 50 Yonsei-ro, Seodaemun-gu, Seoul 03722, Republic of Korea

**Keywords:** endometriosis, adeno-associate virus, RNA interference, gene therapy, endometriosis cell culture, ectopic endometrium

## Abstract

Endometriosis is a chronic estrogen-dependent condition with limited treatment options, often requiring surgery and long-term hormonal therapy that may impair ovarian function. Despite advancements in gene therapy for other diseases, its application in endometriosis remains largely unexplored. This study aimed to evaluate the potential of adeno-associated virus (AAV) vectors for targeted gene therapy in endometriosis. We screened multiple AAV serotypes for infectivity in primary human ectopic and eutopic endometrial cells as well as normal ovarian stromal cells. AAV serotype 3 (AAV3) demonstrated selective infectivity toward endometrial cells while sparing ovarian tissue. AAV3-mediated delivery of small interfering RNA targeting estrogen receptor 2 reduced Estrogen receptor beta (ERβ) expression to 27% in ectopic and 49% in eutopic cells. Under estradiol and inflammatory stimulation, ERβ knockdown led to modest reductions in cellular metabolic activity in eutopic cells, whereas effects in ectopic cells did not reach statistical significance. Dual targeting of ERβ and prostaglandin-endoperoxide synthase 2 (PTGS2) showed numerically lower metabolic activity than controls under some conditions but without consistent statistical significances. These findings suggest that AAV3 can serve as an ovary-sparing, endometriosis-specific vector that facilitates gene silencing while yielding limited phenotypic effects. This gene delivery system may provide a basis for developing future gene-based therapies for endometriosis.

## 1. Introduction

Endometriosis is a chronic gynecological condition characterized by the ectopic presence of endometrial glandular and stromal tissues outside the uterine cavity [1]. It affects approximately 5–15% of women of reproductive age and is a major cause of dysmenorrhea, chronic pelvic pain, and infertility, accounting for up to 50% of infertility cases in affected women [2,3]. Despite the availability of medical treatments including oral contraceptives, progestins, danazol, and gonadotropin-releasing hormone (GnRH) analogs, the recurrence rate remains high—exceeding 50% within five years of treatment cessation [4,5,6,7]. Moreover, current therapies often suppress ovulation or impair ovarian function, limiting their long-term use, particularly in women desiring fertility preservation [8,9]. Therefore, there is an urgent need to develop targeted, long-lasting, and ovary-sparing treatment strategies for endometriosis.

Gene therapy offers a promising approach by delivering therapeutic genes directly into affected tissues [10,11]. While gene therapies have gained clinical success in oncology and genetic disorders, their application to endometriosis remains limited. This gap reflects several challenges, including the lack of suitable vectors for lesion-specific delivery, insufficient validation of molecular targets, and concerns over off-target effects or long-term safety in reproductive tissues. In early preclinical studies, adenoviral vectors delivering angiogenesis inhibitors effectively reduced lesion size in mouse models but caused severe off-target effects, including ovarian atrophy, follicle loss, and reduced hormone production [12]. Similarly, Othman et al. demonstrated that adenovirus-mediated delivery of dominant-negative estrogen receptor constructs suppressed endometriotic cell proliferation and cytokine production, though systemic safety remained a concern [13]. These findings highlight the importance of tissue specificity and vector safety in gene therapy for endometriosis.

Adeno-associated virus (AAV) vectors present a safer and more versatile platform compared with adenoviral vectors, which have raised concerns regarding immunogenicity and ovarian toxicity despite showing efficacy in preclinical models [11]. AAVs are small, non-pathogenic, and minimally immunogenic viruses with serotypes exhibiting distinct tissue tropisms [11]. Unlike adenoviruses, AAVs allow long-term gene expression via episomal maintenance or genome integration and can transduce both dividing and non-dividing cells [14]. In the context of endometriosis, however, no prior studies have explored the suitability of AAV serotypes for selective gene delivery to endometrial tissues. Identifying a serotype that preferentially infects endometriotic lesions while sparing ovarian cells is essential for developing effective and safe gene-based therapies. In parallel, recent advances in local administration techniques—such as sclerotherapy for ovarian endometriomas—have opened potential avenues for future localized gene delivery strategies that may minimize systemic exposure and off-target effects [15].

Endometriosis is increasingly recognized as a complex disease involving not only hormonal and inflammatory dysregulation but also aberrant immune responses, local angiogenesis, and epigenetic alterations. These mechanisms collectively support lesion survival and recurrence and may underlie the limited efficacy of single-target therapies [16]. Estrogen receptor beta (ERβ), encoded by estrogen receptor 2 (ESR2), is significantly overexpressed in ectopic endometrial tissue compared to both eutopic endometrium and estrogen receptor alpha (ERα), suggesting its dominant role in lesion-specific proliferation and immune modulation [16]. Previous studies have demonstrated that ERβ-selective antagonists can suppress ectopic lesion growth in animal models. In addition, the prostaglandin-endoperoxide synthase 2 (PTGS2)-encoding cyclooxygenase-2 (COX-2) is upregulated in endometriotic tissues and contributes to inflammatory signaling and estrogen biosynthesis via the prostaglandin E2–steroidogenic acute regulatory protein (PGE2–StAR) axis [17]. ERβ has been shown to enhance COX-2 expression, while PGE2 further stimulates estrogen synthesis, forming a pathological positive feedback loop [18]. These two genes were selected as dual targets for RNA interference-based therapeutic intervention.

In this study, we aimed to develop an AAV-based gene delivery platform for endometriosis therapy. Specifically, we sought to (1) identify an AAV serotype capable of selectively infecting eutopic and ectopic endometrial cells while sparing normal ovarian stromal cells; (2) evaluate the efficacy of AAV3-mediated small interfering RNA (siRNA) delivery targeting ESR2, PTGS2, or both; (3) quantify gene suppression via quantitative reverse transcription polymerase chain reaction (qRT-PCR); (4) assess the impact on cellular proliferation under hormonal and inflammatory conditions using a cell counting kit-8 (CCK-8) assay. By combining serotype screening and dual-gene targeting in a preclinical model, this study offers a basis for further development of AAV3-based gene therapy approaches for endometriosis.

## 2. Materials and Methods

### 2.1. Study Population and Tissue Collection

Surgical samples from four patients aged over 20 years undergoing laparoscopic ovarian cyst enucleation at Severance Hospital were recruited for this study. Only patients whose pathology confirmed an endometriotic ovarian cyst were included. Patients with borderline or malignant pathology were excluded. A 1 × 1 cm portion of the ovarian endometriotic cyst wall and an endometrial biopsy using a disposable uterine sampler (Rampipella, RI.MOS, Mirandola, Italy) were collected intraoperatively. The number of patients (*n* = 4) was determined by limited tissue availability and variability in primary cell yields.

### 2.2. Primary Cell Culture

#### 2.2.1. Ectopic Endometrial Cell Culture

Biopsied endometriotic cyst linings were transported in phosphate-buffered saline (PBS) and washed repeatedly to remove blood. Tissues were minced and digested in 2 mg/mL collagenase type I (12 mg in Dulbecco’s Phosphate-Buffered Saline (DPBS), 0.25 µm filtered) at 37 °C for 120 min with agitation every 30 min. After digestion, 10 mL of Dulbecco’s Modified Eagle Medium (DMEM)/F12 medium supplemented with 1% penicillin-streptomycin and 10% fetal bovine serum was added. The suspension was filtered through a 100 µm cell strainer and centrifuged at 2000 rpm for 5 min. The cell pellet was resuspended in culture medium and seeded into 75T flasks. After 24 h, non-adherent cells were removed by washing with PBS. Cells were maintained in Ham’s F-10 medium supplemented with 10% fetal calf serum, 2 mmol/L L-glutamine, antibiotics, and 2.5 µg/mL fungizone. Cells were passed upon reaching 90% confluence and fixed with 100% cold methanol for downstream analyses.

#### 2.2.2. Eutopic Endometrial Cell Culture

Uterine endometrial tissues were minced into 1–2 mm^3^ fragments and washed in PBS. Samples were incubated in 5 mL of trypsin- ethylenediaminetetraacetic acid (EDTA) at 37 °C for 40 min with intermittent agitation. The reaction was neutralized with 20 mL of DMEM/F12 medium containing 1% penicillin-streptomycin and 10% fetal bovine serum, then filtered and centrifuged. The pellet was suspended in 5 mL of culture media and seeded in 25T flasks. After 24 h, cells were washed with PBS and cultured under the same conditions as ectopic cells.

### 2.3. AAV Vector Construction and Transduction

#### 2.3.1. AAV Serotype Screening

Self-complementary AAV vectors expressing enhanced green fluorescent protein (EGFP) were packaged into 14 conventional AAV serotypes. These were used to transduce eutopic and ectopic endometrial cells and the human granulosa-lutein 5 (hGL5) ovarian stromal cell line. Cells were plated to achieve 30–50% confluency at the time of transduction. AAV vectors were applied at a fixed multiplicity of infection (MOI; 1 × 10^12^ GC/mL) and incubated overnight, after which the medium was refreshed daily [11]. This MOI was chosen based on manufacturer recommendations and prior literature [19,20]. EGFP expression was analyzed by qRT-PCR and fluorescence microscopy 48 h post-transduction to determine relative infection efficiency and tissue tropism.

#### 2.3.2. siRNA Design and AAV Vector Construction

**Figure 1:**
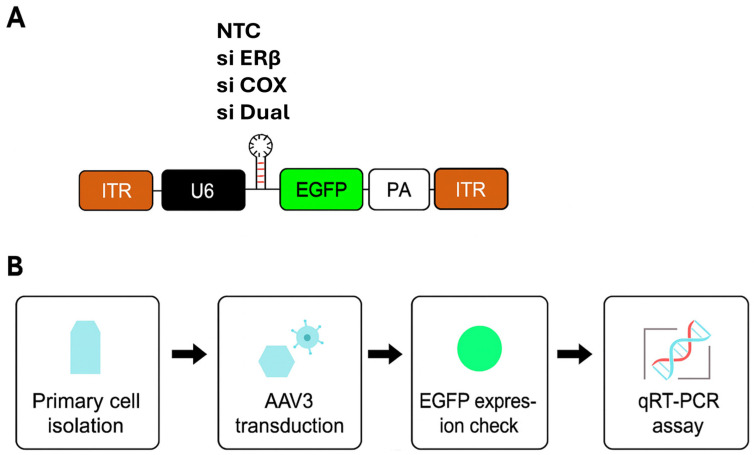
Schematic representation of the AAV3 vector backbone and experimental workflow. (**A**) AAV3 backbone design. All constructs contained inverted terminal repeats (ITRs) flanking a U6 promoter-driven siRNA cassette (NTC, siERβ, siCOX2, or siERβ + siCOX2, hereafter siDual) and a CMV promoter-driven EGFP reporter for tracking transduction efficiency, followed by a polyadenylation signal (PA). The NTC construct was used as the non-targeting control. (**B**) Experimental workflow. Primary endometrial cells were isolated and transduced with AAV3 vectors, followed by confirmation of EGFP expression and subsequent analyses including qRT-PCR and proliferation assays. AAV: adeno-associated virus; Erβ: Estrogen receptor β; COX2: cy-clooxygenase-2; CMV: cytomegalovirus; EGFP: enhanced green fluorescent protein; si Dual: siERβ + siCOX2; NTC: non-targeting siRNA cassette; qRT-PCR: quantitative reverse transcription polymerase chain reaction.

The siRNA sequences targeting ESR2 were pre-designed and cloned into AAV3 vectors for delivery. The PTGS2-targeting siRNA was obtained as a premade construct from VectorBuilder (VectorBuilder Inc., Chicago, IL, USA). As a control, we used a non-targeting siRNA cassette cloned into the same AAV3 backbone, hereafter referred to as NTC. In all constructs, siRNA expression was driven by a U6 promoter, and EGFP expression was under the control of a cytomegalovirus (CMV) promoter for infection tracking. For simplicity, groups are referred to by their siRNA designation (NTC, siERβ, siCOX2, siERβ + siCOX2 [hereafter referred to as siDual]) throughout the manuscript, but all vectors were AAV3-based. A schematic of the vector backbone and experimental workflow is provided in Figure 1. To identify an effective ESR2-targeting sequence, three premade siRNAs (ID: 145909, 145910, 145911; VectorBuilder) were screened in ectopic endometrial cells. (Supplementary Table S1) Among these, siRNA 145911 showed the most efficient downregulation of ESR2 mRNA and was therefore selected for subsequent experiments.

### 2.4. Fluorescent Imaging

At 48 h post-transduction, cells were fixed with 4% paraformaldehyde for 15 min at room temperature and washed with PBS. Coverslips were mounted with DAPI-containing antifade medium (Vector Laboratories, Burlingame, CA, USA). Fluorescence images were obtained using an inverted fluorescence microscope (Axio Observer, Carl Zeiss, Germany) and analyzed with ZEN software (ZEISS, Oberkochen, Germany, version 3.4). Identical exposure settings were used across samples.

### 2.5. RNA Extraction and qRT-PCR

Total RNA was extracted from cells using TRIzol^®^ Reagent (Thermo Fisher Scientific, Waltham, MA, USA). Chloroform extraction was followed by ethanol precipitation, and RNA was purified using spin cartridges with sequential wash buffers. RNA was eluted in RNase-free water and stored at −80 °C. Gene expression of ESR2 and PTGS2 was quantified using SYBR Green-based qRT-PCR. All target gene expression levels were normalized to glyceraldehyde-3-phosphate dehydrogenase as the housekeeping reference gene. Relative expression levels were calculated using the ΔΔCt method [21].

### 2.6. Cell Proliferation/Metabolic Activity Assay Under Hormonal and Inflammatory Conditions

Cell viability and metabolic activity were assessed using the CCK-8 assay (Dojindo Molecular Technologies, Kumamoto, Japan). Primary ectopic and eutopic endometrial cells were seeded at a density of 1000 cells per well in 96-well plates and transduced with one of the following AAV3 constructs: NTC, siERβ, siCOX2, or si Dual vectors. Although CCK-8 is widely used as a surrogate for proliferation, it primarily measures cellular dehydrogenase activity and metabolic viability and therefore reflects metabolic activity that may correlate with—but does not exclusively represent—cellular proliferation.

Two independent experiments were conducted to evaluate the functional impact of gene knockdown under hormonal and inflammatory conditions. In the first experiment, all four transduction groups were cultured under two media conditions: (1) standard growth medium (control) and (2) medium supplemented with 1 nM estradiol and 10 nM prostaglandin E2 (PGE2). This allowed direct comparison of hormone/inflammation-induced proliferation across vector groups. In the second experiment, only the NTC and si Dual vector groups were evaluated. Cells were cultured under four conditions: (1) control medium, (2) 1 nM estradiol alone, (3) 10 nM PGE2 alone, and (4) 1 nM estradiol + 10 nM PGE2. This setup was designed to isolate the specific effects of individual and combined hormonal/inflammatory stimuli on proliferation in dual targeting versus control settings.

After 48 h of stimulation, 10 µL of CCK-8 solution was added to each well and incubated for 2 h. Absorbance at 450 nm was measured using a microplate reader. All experiments were performed in technical triplicates, and values were expressed as mean ± standard deviation (SD). While tissues were obtained from four independent patients, raw donor-level datasets could not be retained due to limitations in tissue yields and laboratory resources, preventing systematic biological replication.

### 2.7. Statistical Analysis

All statistical analyses and figure generation were performed using Python (version 3.10.12), utilizing the Pingouin (version 0.5.3) and Matplotlib libraries (version 3.7.2). For comparisons between two groups (e.g., control vs. siERβ), unpaired two-tailed Student’s t-tests were applied. For comparisons involving more than two groups (e.g., NTC, siERβ, siCOX2, siDual), one-way ANOVA followed by Tukey’s HSD post hoc test was conducted using Pinguin’s pairwise Tukey function. A *p*-value less than 0.05 was considered statistically significant.

All data are presented as mean ± SD. Each experiment was performed in technical triplicate only, without biological (donor-level) replication, and therefore results should be interpreted as exploratory. Effect size estimates (Cohen’s d for pairwise comparisons) were additionally calculated and are reported in Supplementary Table S2 to provide an indication of the magnitude of observed differences. Sample size was not predetermined by statistical power analysis, as this study was exploring and conducted in vitro.

### 2.8. Ethics Statement

Human endometrial and endometriotic tissues were obtained with written informed consent under protocols approved by the Institutional Review Board of Severance Hospital, Yonsei University College of Medicine (IRB No. 4-2018-1019, approved on 17 December 2018).

## 3. Results

### 3.1. AAV Serotype Screening in Endometrial and Ovarian Cells

**Figure 2:**
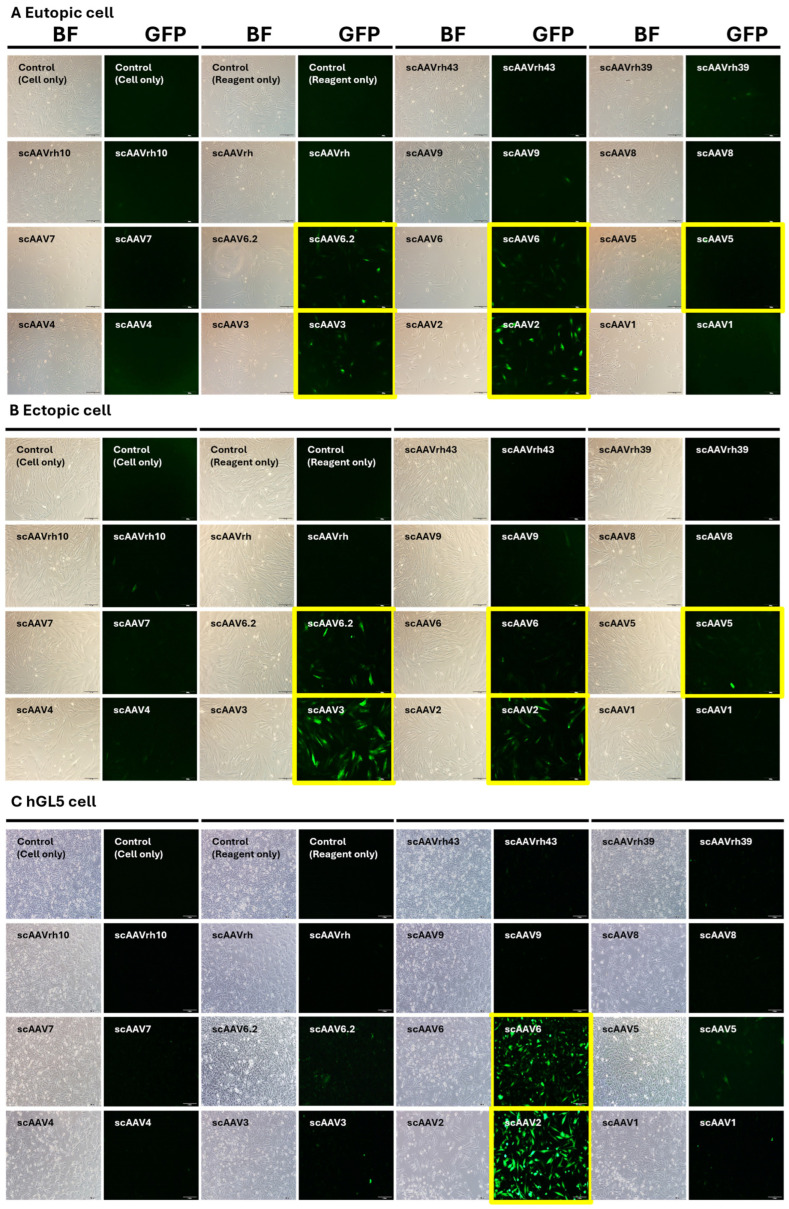
AAV serotype screening in eutopic and ectopic endometrial cells and ovarian stromal cells. (**A**) Fluorescence microscopy images of eutopic cells transduced with EGFP-expressing AAV serotypes. (**B**) Corresponding images from ectopic cells. (**C**) Images of hGL5 ovarian stromal cells under the same conditions. Robust EGFP expression was observed with scAAV6.2, 6, 5, 3, and 2 in eutopic and ectopic cells, while scAAV3 showed minimal fluorescence in ovarian stromal cells, indicating tissue-specific transduction. Yellow boxes indicate the serotype with highest infectivity for each respective cell type. Quantitative qRT-PCR results of EGFP expression across serotypes are provided in Figure 3 and Supplementary Table S3, further supporting the selective tropism of AAV3. AAV: adeno-associated virus; BF: bright field; EGFP: enhanced green fluorescent protein; hGL5: human granulosa-lutein 5; qRT-PCR: quantitative reverse transcription polymerase chain reaction.

**Figure 3:**
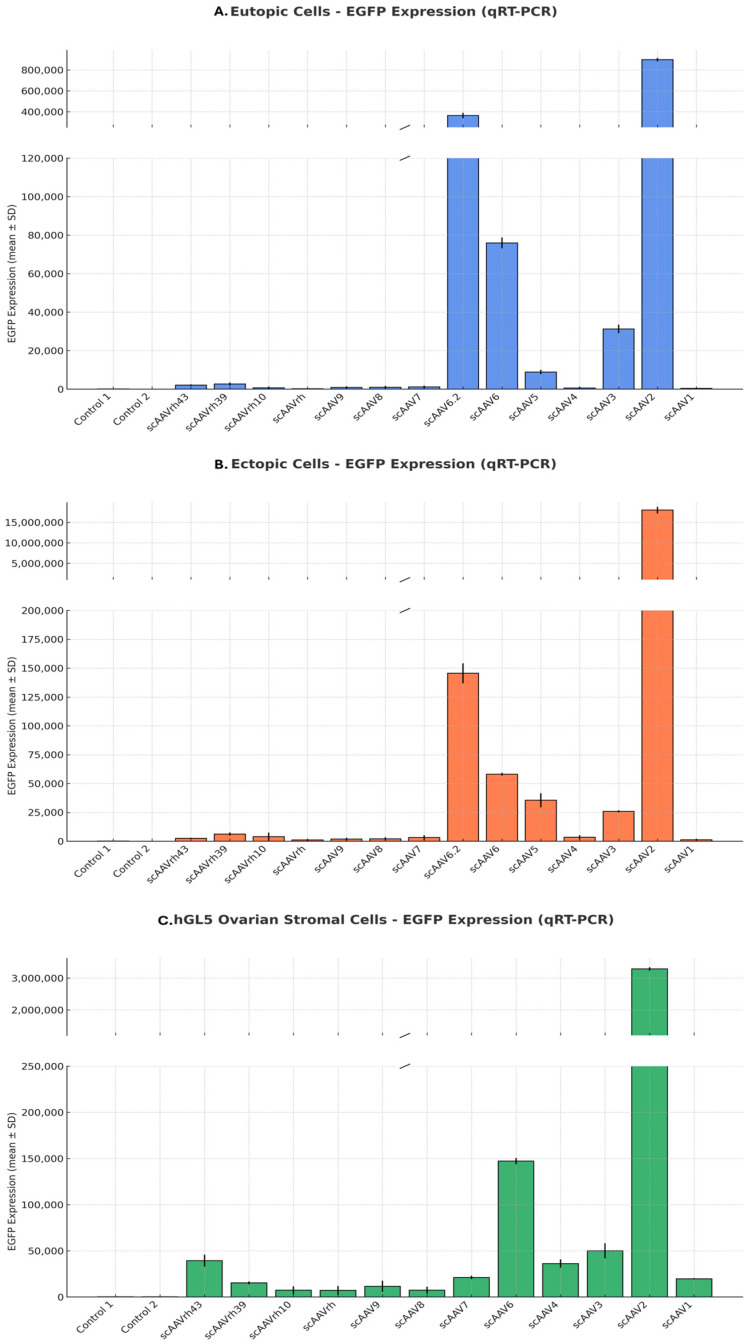
Quantitative assessment of AAV serotype transduction efficiency in endometrial and ovarian cells. Relative EGFP expression levels were measured by qRT-PCR at 48 h after transduction with self-complementary AAV serotypes (1 × 10^12^ GC/mL). Data is presented as mean ± SD from technical triplicates. (**A**) Eutopic endometrial stromal cells. (**B**) Ectopic endometrial stromal cells. (**C**) hGL5 ovarian stromal cells. Broken y-axes are used to visualize both low- and high-expression serotypes within the same scale. Among the tested serotypes, scAAV3 demonstrated strong transduction in eutopic and ectopic cells, whereas scAAV2 showed the highest expression in ovarian stromal cells. These results support AAV3 as a selective candidate vector for ovary-sparing gene delivery in endometriosis. Data is shown as mean ± SD from technical triplicates. AAV: adeno-associated virus; EGFP: enhanced green fluorescent protein; hGL5: human granulosa-lutein 5; qRT-PCR: quantitative reverse transcription polymerase chain reaction.

To determine the optimal AAV serotype for targeted gene delivery in endometriosis, we transduced 14 conventional AAV serotypes, each encoding an EGFP reporter gene, into primary cultures derived from eutopic and ectopic endometrial tissues, as well as the hGL5 ovarian stromal cell line. (Figure 2, Supplementary Figure S1) Fluorescence microscopy revealed consistently high EGFP expression in eutopic and ectopic endometrial cells infected with scAAV6.2, 6, 5, 3, and 2, both in tissue sections and cultured cells. In contrast, hGL5 cells exhibited strong EGFP expressions only with scAAV6.2, 6, 5, and 2, whereas AAV3-transduced cells showed minimal fluorescence. These findings were further supported by qRT-PCR quantification of EGFP expression, showing that AAV3 induced markedly higher transgene expression in eutopic and ectopic endometrial cells while expression in ovarian stromal cells remained negligible (Figure 3 and Supplementary Table S3). Based on these results, AAV3 was selected as the delivery vector for subsequent experiments due to its preferential transduction efficiency in endometrial tissues while sparing ovarian stromal cells.

### 3.2. AAV3-Mediated ERβ Suppression in Endometrial Cells

**Figure 4:**
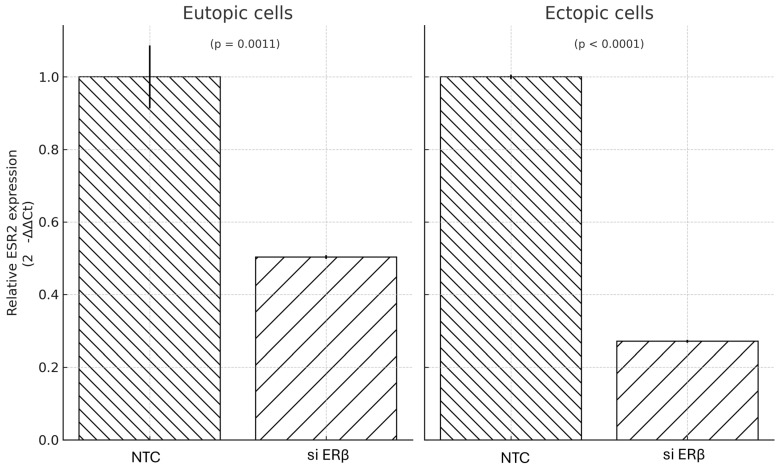
Relative ESR2 (ERβ) mRNA expression following AAV3-mediated siERβ delivery in eutopic and ectopic endometrial cells. Significant downregulation was observed in both cell types compared to controls (*p* = 0.0011 for eutopic, *p* < 0.0001 for ectopic). Data is shown as mean ± SD from technical triplicates. AAV: adeno-associated virus; ESR2: Estrogen receptor 2; Erβ: Estrogen receptor β; NTC: non-targeting siRNA cassette.

Primary eutopic and ectopic endometrial cells were transduced with siERβor NTC. Successful infection was confirmed by GFP fluorescence imaging, which demonstrated widespread intracellular delivery of the recombinant vector in both cell types. Quantitative RT-PCR analysis showed that ERβ expression was significantly reduced following siERβ transduction, with mRNA levels decreased to 49% of control values in eutopic cells (*p* = 0.0011) and 27% in ectopic cells (*p* = 0.000015), indicating statistically significant knockdown in both cell types (Figure 4).

### 3.3. Comparative Effects of Single and Dual Gene Silencing on Cell Viability/Metabolic Activity Under Hormonal and Inflammatory Conditions

**Figure 5:**
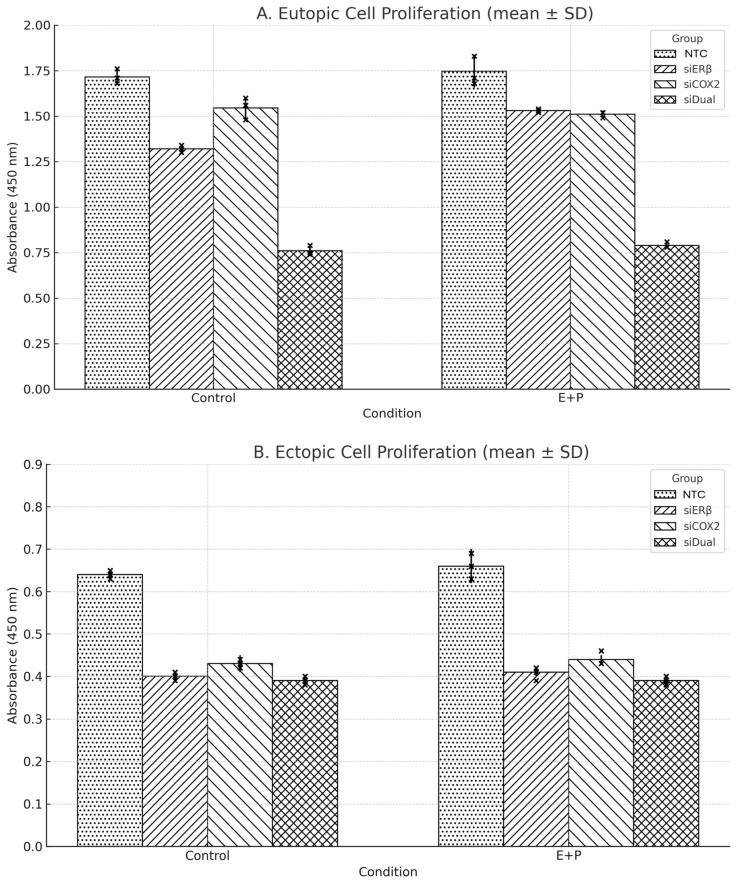
Cell viability/metabolic activity (CCK-8) after siRNA-AAV3 transduction under control vs. E + P stimulation. (**A**) In eutopic cells, siCOX2 and siDual significantly reduced proliferation (*p* = 0.006 and *p* = 0.035, respectively). Tukey’s post hoc analysis further confirmed significantly lower values in all knockdown groups compared with NTC under E + P (all *p* < 0.001). (**B**) In ectopic cells, within-group changes were not significant (all *p* > 0.3), but Tukey’s analysis indicated that siERβ, siCOX2, and siDual groups were significantly lower than NTC under E + P (all *p* < 0.001). Data is shown as mean ± SD from technical triplicates. AAV: adeno-associated virus; ESR2: Estrogen receptor 2; Erβ: Estrogen receptor β; COX2: cyclooxygenase-2; CCK-8: cell counting kit-8; si Dual: siERβ + siCOX2; E + P: 1 nM estradiol + 10 nM PGE2; NTC: non-targeting siRNA cassette.

Cell viability/metabolic activity was assessed using the CCK-8 assay after siRNA-AAV3 transduction under estradiol and prostaglandin E2 (E + P) stimulation (Figure 5). In eutopic cells, absorbance values in the NTC group showed a slight increase from 1.74 to 1.78. The siERβ group exhibited a small decrease (1.30 to 1.28), while siCOX2 showed a modest reduction (1.55 to 1.48; *p* = 0.006). siDual also resulted in a decrease (0.78 to 0.76; *p* = 0.035). Tukey’s post hoc analysis under E + P stimulation confirmed that siERβ, siCOX2, and siDual groups all showed significantly lower absorbance compared with NTC (all *p* < 0.001). In ectopic cells, absorbance changes were minimal across groups, with no statistically significant differences observed (all *p* > 0.3). However, under E + P stimulation, Tukey’s post hoc analysis again demonstrated significant reductions in absorbance for all knockdown groups compared with NTC (all *p* < 0.001).

### 3.4. Dual Targeting of ERβ and COX-2 Under Hormonal and Inflammatory Conditions: Effects on Cell Viability/Metabolic Activity

**Figure 6:**
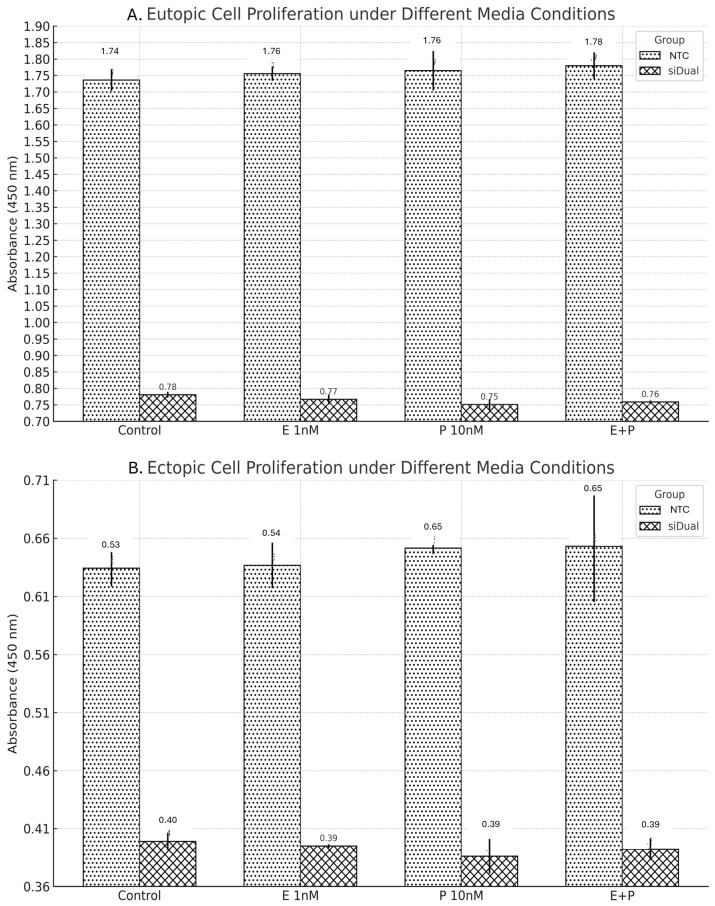
Cell viability/metabolic activity (CCK-8) after dual gene suppression under various media conditions. (**A**) Eutopic and (**B**) ectopic endometrial cells transduced with siDual or NTC were cultured under control, estradiol (E), prostaglandin E_2_ (P), or combined (E + P) conditions. No significant differences were observed across media within each group (all *p* > 0.05), but siDual values were consistently lower than NTC under all conditions (all *p* < 0.001). Data is shown as mean ± SD from technical triplicates. AAV: adeno-associated virus; ESR2: Estrogen receptor 2; Erβ: Estrogen receptor β; COX2: cyclooxygenase-2; CCK-8: cell counting kit-8; GFP: green fluorescent protein; si Dual: siERβ + siCOX2; E + P: 1 nM estradiol + 10 nM PGE2; NTC: non-targeting siRNA cassette.

To evaluate the effects of ERβ and COX-2 gene suppression under various hormonal and inflammatory conditions, cell proliferation was assessed after transduction with siDual or NTC vectors across four media conditions: control, estradiol (E 1nM), prostaglandin E2 (P 10 nM), and combined E + P stimulation (Figure 6). In eutopic cells, absorbance values in the NTC group were 1.74 (control), 1.76 (E), 1.76 (P), and 1.78 (E + P), Corresponding siDual group values were 0.78, 0.77, 0.75, and 0.76. Although the siDual group showed consistently lower absorbance compared with NTC, one-way ANOVA revealed no statistically significant differences across conditions (*p* = 0.639 for NTC; *p* = 0.079 for siDual). Between-group comparisons indicated that siDual was significantly lower than NTC under all conditions (all *p* < 0.001). In ectopic cells, absorbance values were similarly stable across conditions, with NTC at 0.63–0.65 and siDual at 0.39–0.40. ANOVA again showed no significant differences within groups (*p* = 0.762 for NTC; *p* = 0.469 for siDual). However, siDual was consistently lower than NTC under every condition tested (all *p* < 0.001). Effect size estimates (Cohen’s d) for each knockdown group relative to the NTC are summarized in Supplementary Table S2.

## 4. Discussion

This study aimed to develop a targeted, ovary-sparing gene delivery platform for endometriosis using an AAV-based approach. Among various serotypes tested, AAV3 exhibited a favorable transduction profile by efficiently infecting both eutopic and ectopic endometrial cells while exhibiting lower transduction efficiency in ovarian stromal cells. This selective tropism makes AAV3 a promising candidate for localized gene therapy applications, particularly where ovarian preservation is essential. These findings are consistent with previous literature highlighting the tissue-specific transduction patterns of AAV serotypes [11,22].

Given the estrogen-dependent and inflammatory nature of endometriosis, we explored two key molecular targets: ERβ and PTGS2. ERβ, known to be overexpressed in ectopic lesions compared to eutopic endometrium and ERα, has been implicated in lesion proliferation and immune evasion [18]. PTGS2, encoding COX-2, mediates prostaglandin E2 production and promotes estrogen biosynthesis via the StAR pathway, further fueling endometriotic growth [17].

In our study, gene silencing of ERβ via siRNA-AAV3 vectors was confirmed at the mRNA level, with greater suppression observed in ectopic cells compared to eutopic cells. However, this molecular suppression did not translate into significant differences in cell viability/metabolic activity under estradiol and inflammatory stimulation. Across both eutopic and ectopic cells, neither ERβ nor COX-2 single targeting, nor dual targeting, resulted in statistically significant reductions in cell viability/metabolic activity. Post hoc Tukey’s tests confirmed that, although siCOX2 and siDual showed lower metabolic activity compared with the NTC group under E + P stimulation, the overall effects were modest and should be interpreted cautiously in this exploratory context. The magnitude of phenotypic suppression was modest despite robust gene knockdown of ERβ. This discrepancy suggests that additional signaling mechanisms beyond ERβ and COX-2 are involved in maintaining the proliferative phenotype of endometriotic cells.

This observation is consistent with prior animal studies where ERβ antagonism led to partial reduction in lesion growth but failed to completely inhibit proliferation [18,23]. Redundant and compensatory pathways—such as PI3K/AKT, MAPK/ERK, NF-κB, and WNT/β-catenin—have been implicated in endometriotic cell survival, independent of estrogen or prostaglandin signaling [16,24,25,26,27]. These alternative routes may buffer the effects of targeted gene silencing, limiting the impact on downstream proliferation.

Similarly, in our dual-targeting experiments, COX-2 silencing alone reduced proliferation, and only a slight additive effect was observed with combined targeting, particularly in ectopic cells. This limited response further supports the notion that multiple redundant pathways are active in endometriotic cells, requiring broader or alternative combinational strategies to achieve robust suppression [16]. Taken together, our findings suggest—but do not confirm—that combination or multi-targeted approaches may be required to more effectively suppress proliferation in endometriotic cells. This interpretation is consistent with the exploratory ANOVA/Tukey analyses, which indicated group-level differences under E + P but did not reveal broad or consistent effects across all conditions.

Additionally, this study was conducted in vitro using primary cells derived from a limited number of patients (n = 4), which may not fully capture the molecular and cellular heterogeneity of endometriotic lesions. The results may therefore be influenced by inter-individual variability, and further validation in a larger sample set is warranted. While ESR2 mRNA suppression confirmed efficient ERβ silencing, corresponding suppression of PTGS2 mRNA was not assessed due to technical constraints, limiting our ability to evaluate the specific contribution of COX-2 knockdown to the observed effects. In addition, protein-level validation of ERβ and COX-2 suppression (e.g., by Western blot or immunofluorescence) and functional assays such as PGE2 quantification were not performed. These analyses will be critical in future studies to comprehensively validate the molecular and functional impact of target gene silencing. Because raw donor-level data could not be retained, technical triplicates were analyzed, and effect size estimates (Cohen’s d) are provided in Supplementary Table S2 as an exploratory measure only, rather than a definitive indicator of biological effect.

Another methodological limitation is that cell viability/metabolic activity was assessed solely by the CCK-8 assay, which measures metabolic activity rather than direct cell division. As such, the observed decreases may reflect cytostatic or cytotoxic effects. Orthogonal proliferation and apoptosis assays (e.g., EdU/BrdU incorporation, Ki-67 staining, Annexin V/PI flow cytometry) will be required in future studies to clarify whether AAV3-mediated gene silencing primarily suppresses cell proliferation or induces cell death. Baseline differences between siRNA groups, particularly in eutopic control conditions, may reflect vector-related effects such as MOI load, seeding variability, or cassette-associated toxicity. In this study, all transductions were performed at the manufacturer-recommended MOI of 1 × 10^12^ GC/mL, a dosage supported by prior literature and supplier guidelines [19,20]. However, systematic MOI titration was not undertaken, and we therefore cannot fully exclude vector-related contributions to the observed baseline differences. Future studies incorporating titration and additional vector controls will be important to rigorously confirm that the observed effects are specific to siRNA-mediated knockdown. In addition, our serotype screening did not include FACS-based quantification, MOI titration, or long-term time-course analyses. While qRT-PCR provides supportive data, more rigorous approaches will be needed in future work.

In considering translational applications, vector safety is an important factor. While adenoviral vectors have demonstrated efficacy in preclinical models of endometriosis, their use has raised safety concerns, including strong innate immune activation, systemic inflammatory responses, and ovarian toxicity such as follicle depletion and impaired hormone production [12,13]. By contrast, AAV vectors are replication-deficient, generally exhibit lower immunogenicity, and usually persist as episomes rather than integrating into the host genome. These features contribute to a more favorable safety profile, as also reflected in the clinical approval of several AAV-based therapies (e.g., Luxturna, Zolgensma). Taken together, AAVs may therefore represent a safer and more sustainable platform compared with adenoviral vectors for developing ovary-sparing gene therapy approaches in endometriosis.

Moreover, although gene silencing was clearly achieved at the transcript level, the impact on cell proliferation was modest and did not reach statistical significance in most conditions. This disparity implies that multiple redundant signaling pathways may compensate for ERβ and COX-2 suppression, necessitating exploration of additional or synergistic targets to achieve more robust phenotypic effects. Future studies should consider targeting other signaling cascades such as NF-κB, WNT/β-catenin, or VEGF pathways, which have been implicated in lesion growth and persistence [27].

Importantly, the in vivo pharmacokinetics, biodistribution, and long-term transgene expression stability of AAV3 vectors remain to be established. Immune responses to the viral capsid or repeated administration may limit therapeutic efficacy, and these challenges must be addressed in preclinical models. Off-target effects in non-endometrial tissues, particularly under systemic exposure, also require evaluation. Localized delivery approaches such as ultrasound-guided sclerotherapy—which are already used in clinical settings for treating endometriomas—may offer a feasible route for administering AAV3-based therapeutics while minimizing systemic exposure and off-target effects [15,28].

Despite these limitations, our findings provide initial proof-of-concept that AAV3 may serve as a selective and ovary-sparing gene delivery vehicle in endometriosis, offering a rationale for further preclinical investigation rather than immediate translation.

## 5. Conclusions

In conclusion, this study provides initial evidence supporting the use of AAV3-based vectors for dual-targeted gene therapy in endometriosis. While ERβ-targeted siRNA delivery resulted in efficient gene knockdown, the phenotypic impact on cell viability/metabolic activity was limited, and dual targeting with COX-2 did not yield additive effects. These results highlight the complexity and redundancy of proliferative signaling in endometriotic cells. Future studies incorporating additional targets, in vivo validation, and optimized delivery strategies, including localized therapeutic approaches, will be essential to advance this platform toward clinical application.

## Data Availability

The original contributions presented in this study are included in the article/Supplementary Material. Further inquiries can be directed to the corresponding author.

## Supplementary Materials

The following supporting information can be downloaded at: https://www.mdpi.com/article/10.3390/microorganisms13092144/s1, Figure S1: AAV serotype transduction in eutopic and ectopic endometrial tissue sections; Table S1: ESR2-targeting siRNA sequences screened in ectopic endometrial cells; Table S2: Cohen’s d effect size estimates for proliferation assays; Table S3: qRT-PCR quantification of EGFP expression across AAV serotypes; Table S4: Raw CCK-8 proliferation assay data; File S1: Python script for reproducibility.py.
