# Supplementary material for: Identification of Adeno-Associate Virus (AAV) Serotype for Endometriosis Therapy and Effect of AAV-Mediated RNAi Delivery on Gene Expression and Cell Proliferation in In Vitro Endometrial Cell Culture
Source: Microorganisms. 2025 Sep 13;13(9):2144. doi: 10.3390/microorganisms13092144 (PMC12472493; doi:10.3390/microorganisms13092144)
Supplement: Supplementary file 1 [file microorganisms-13-02144-s001.zip › Figure S1.pdf]

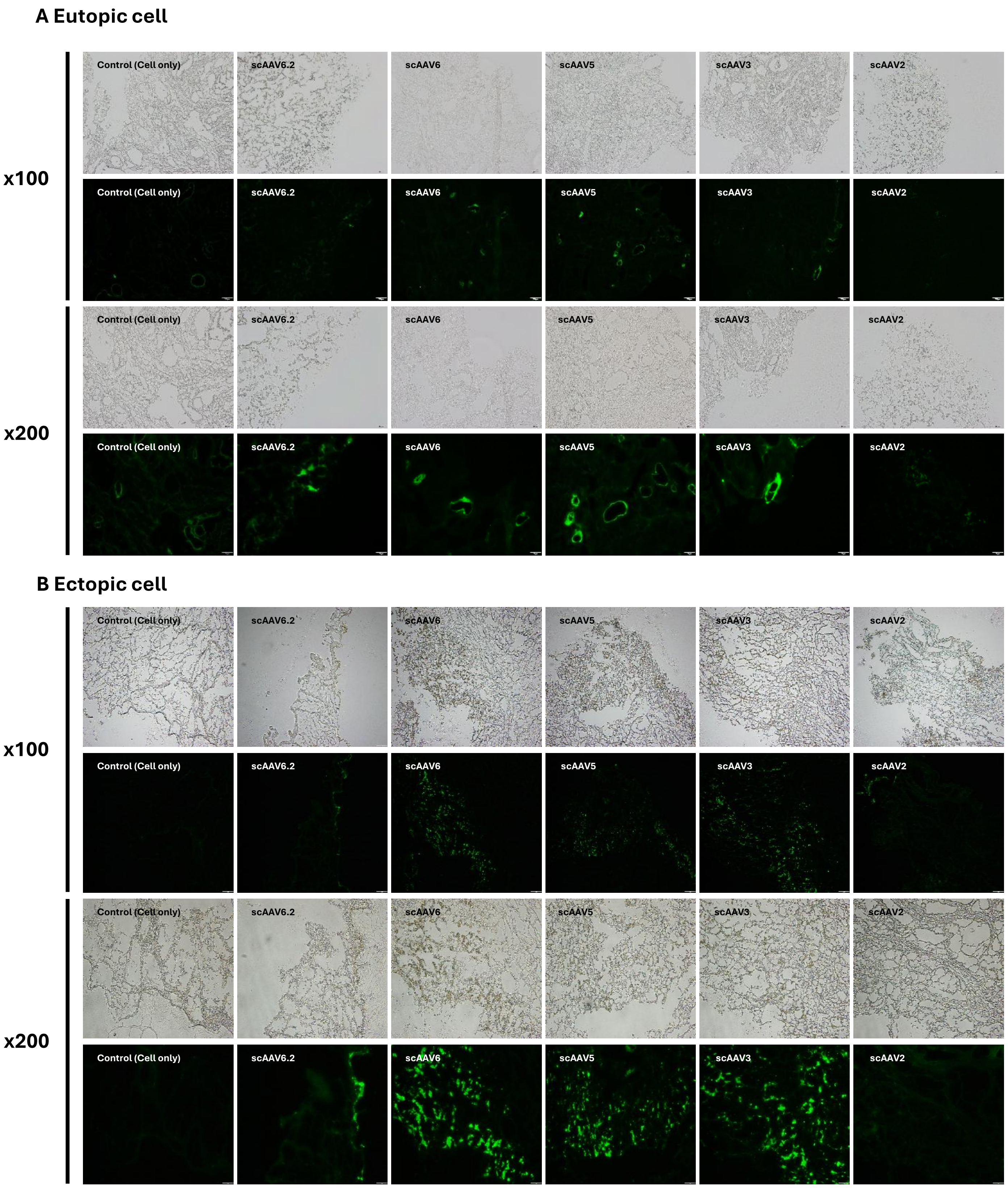

Supplementary Figure S1. AAV serotype transduction in eutopic and ectopic endometrial tissue sections. Representative fluorescence microscopy images of eutopic and ectopic endometrial tissue sections transduced with EGFP-expressing AAV vectors (scAAV6.2, 6, 5, 3, and 2), compared to non-transduced control.
Images were captured at both 100× and 200× magnifications. Robust EGFP expression was observed in multiple serotypes, with scAAV3 showing strong expression in endometrial tissue consistent with in vitro findings.
