# Supplementary material for: Identification of Adeno-Associate Virus (AAV) Serotype for Endometriosis Therapy and Effect of AAV-Mediated RNAi Delivery on Gene Expression and Cell Proliferation in In Vitro Endometrial Cell Culture
Source: Microorganisms. 2025 Sep 13;13(9):2144. doi: 10.3390/microorganisms13092144 (PMC12472493; doi:10.3390/microorganisms13092144)
Supplement: Supplementary file 1 [file microorganisms-13-02144-s001.zip › Supplementary File S1.docx]

# S1_python_analysis.py

# Reproducible analysis for Baek et al. (Endometriosis AAV study)

#

# This script reproduces the statistical analyses and figures corresponding to:

# - Results 3.3 -> now Figure 5 (Exp1: control vs E+P; siGFP vs siERβ/siCOX2/siDual)

# - Results 3.4 -> now Figure 6 (Exp2: control vs E vs P vs E+P; within siGFP or siDual)

#

# Inputs:

# Excel workbook with multiple sheets, each sheet named like:

# "eutopic cell siGFP", "eutopic cell siERb", "eutopic cell siCox2", "eutopic cell siERb+Cox2",

# "ectopic cell siGFP", "ectopic cell siERb", "ectopic cell si Cox2", "ectopic cell si ERb+cox2", ...

# Each sheet contains columns:

# Condition | Raw1 | Raw2 | Raw3 | Calculated_Mean | Calculated_SD

#

# Libraries (versions used in manuscript):

# pandas==2.2.*, numpy==1.26.*, pingouin==0.5.3, matplotlib==3.8.*

from pathlib import Path

import re

import numpy as np

import pandas as pd

import matplotlib.pyplot as plt

import pingouin as pg

# ----------------------------

# Paths

# ----------------------------

DATA_XLSX = Path("Table S4 Raw CCK-8 proliferation assay data.xlsx") #

OUTDIR = Path("analysis_outputs")

OUTDIR.mkdir(exist_ok=True)

# ----------------------------

# Helpers

# ----------------------------

def norm_cell_type(name: str) -> str:

s = name.lower()

if "eutopic" in s:

return "eutopic"

if "ectopic" in s:

return "ectopic"

return "unknown"

def norm_group(name: str) -> str:

s = name.lower()

# tolerate spacing/case variants

if "sigfp" in s or "si gfp" in s or "gfp" in s:

return "siGFP"

if "sier" in s or "si er" in s: # siERb/siERβ

if "+" in s:

return "siDual"

return "siERβ"

if "cox2" in s or "co x2" in s or "si cox" in s:

if "+" in s:

return "siDual"

return "siCOX2"

if "dual" in s or ("sier" in s and "cox" in s):

return "siDual"

return "unknown"

def norm_condition(x: str) -> str:

if not isinstance(x, str):

return str(x)

s = x.strip().lower()

# normalize common labels

if s.startswith("con"):

return "control"

if s.startswith("e ") or s == "e" or "estradiol" in s:

# handle "e 1nm"

return "E"

if s.startswith("p ") or s == "p" or "pge2" in s:

return "P"

if "+" in s or "e+p" in s:

return "E+P"

return x

def read_sheet(xls: pd.ExcelFile, sheet_name: str) -> pd.DataFrame:

df = pd.read_excel(xls, sheet_name=sheet_name)

# standardize columns

colmap = {}

for c in df.columns:

cl = str(c).strip().lower().replace("’","'")

if cl.startswith("cond"):

colmap[c] = "Condition"

elif cl == "raw1" or "raw 1" in cl:

colmap[c] = "Raw1"

elif cl == "raw2" or "raw 2" in cl:

colmap[c] = "Raw2"

elif cl == "raw3" or "raw 3" in cl:

colmap[c] = "Raw3"

elif "calculated_mean" in cl or (cl == "mean") or ("calc" in cl and "mean" in cl):

colmap[c] = "Calculated_Mean"

elif "calculated_sd" in cl or (cl == "sd") or ("calc" in cl and "sd" in cl):

colmap[c] = "Calculated_SD"

df = df.rename(columns=colmap)

# keep required columns only if present

needed = {"Condition","Raw1","Raw2","Raw3"}

if not needed.issubset(df.columns):

raise ValueError(f"Missing required columns in sheet {sheet_name}: {df.columns}")

# coerce numerics

for c in ["Raw1","Raw2","Raw3","Calculated_Mean","Calculated_SD"]:

if c in df.columns:

df[c] = pd.to_numeric(df[c], errors="coerce")

# normalize condition

df["Condition"] = df["Condition"].astype(str).apply(norm_condition)

return df

def expand_triplicates(df: pd.DataFrame, cell_type: str, group: str, sheet: str) -> pd.DataFrame:

"""Return long df with columns: cell_type, group, condition, replicate, value"""

rows = []

for _, r in df.iterrows():

cond = r["Condition"]

for i, col in enumerate(["Raw1","Raw2","Raw3"], start=1):

val = r[col]

if pd.notna(val):

rows.append({"cell_type": cell_type, "group": group,

"condition": cond, "replicate": i,

"value": float(val), "sheet": sheet})

return pd.DataFrame(rows)

def label_experiment(conds: list[str]) -> str:

"""Exp1: ['control','E+P'] (Results 3.3 -> Figure 5)

Exp2: ['control','E','P','E+P'] (Results 3.4 -> Figure 6)

"""

s = sorted(set(conds))

if s == ["E+P","control"]:

return "exp1"

if s == ["E","E+P","P","control"]:

return "exp2"

# fallback: infer by count

if len(s) == 2:

return "exp1"

if len(s) == 4:

return "exp2"

return "unknown"

# ----------------------------

# Load all sheets -> long format

# ----------------------------

xls = pd.ExcelFile(DATA_XLSX)

all_long = []

meta = [] # store per-sheet meta (cell_type, group, experiment)

for sh in xls.sheet_names:

cell_t = norm_cell_type(sh)

group = norm_group(sh)

try:

df = read_sheet(xls, sh)

except Exception as e:

print(f"[WARN] Skipping sheet '{sh}': {e}")

continue

exp = label_experiment(df["Condition"].tolist())

long_df = expand_triplicates(df, cell_t, group, sh)

if long_df.empty:

print(f"[WARN] No data in sheet '{sh}'.")

continue

long_df["experiment"] = exp

all_long.append(long_df)

meta.append({"sheet": sh, "cell_type": cell_t, "group": group,

"experiment": exp, "conditions": sorted(df["Condition"].unique().tolist())})

if not all_long:

raise SystemExit("No usable sheets found. Please check sheet names/columns.")

long = pd.concat(all_long, ignore_index=True)

pd.DataFrame(meta).to_csv(OUTDIR/"sheet_meta_overview.csv", index=False)

# ----------------------------

# Results 3.3 (now Figure 5): Welch t-test + Cohen's d

# ----------------------------

def run_exp1_stats(long_df: pd.DataFrame) -> pd.DataFrame:

res = []

df1 = long_df[long_df["experiment"] == "exp1"] # control vs E+P

if df1.empty:

print("[INFO] No Exp1 data detected.")

return pd.DataFrame()

for cell in sorted(df1["cell_type"].unique()):

for cond in sorted(df1["condition"].unique()):

sub = df1[(df1.cell_type==cell) & (df1.condition==cond)]

if sub.empty:

continue

base = sub[sub.group=="siGFP"]["value"]

for grp in ["siERβ","siCOX2","siDual"]:

comp = sub[sub.group==grp]["value"]

if base.empty or comp.empty:

continue

# Welch t-test

tt = pg.ttest(base, comp, paired=False, alternative="two-sided")

# Cohen's d

d = pg.compute_effsize(base, comp, eftype="cohen")

res.append({

"experiment": "exp1", "figure": "Fig5",

"cell_type": cell, "condition": cond, "comparison": f"{grp} vs siGFP",

"t": tt["T"].iloc[0], "df": tt["dof"].iloc[0],

"p": tt["p-val"].iloc[0], "cohens_d": d

})

out = pd.DataFrame(res)

if not out.empty:

out.to_csv(OUTDIR/"Fig5_Res33_ttests_effectsizes.csv", index=False)

return out

# ----------------------------

# Results 3.4 (now Figure 6): ANOVA + Tukey HSD

# ----------------------------

def run_exp2_anova_tukey(long_df: pd.DataFrame):

df2 = long_df[long_df["experiment"] == "exp2"] # control, E, P, E+P

if df2.empty:

print("[INFO] No Exp2 data detected.")

return

for cell in sorted(df2["cell_type"].unique()):

for grp in ["siGFP","siDual"]:

sub = df2[(df2.cell_type==cell) & (df2.group==grp)]

if sub.empty:

continue

# One-way ANOVA across conditions

aov = pg.anova(dv="value", between="condition", data=sub, detailed=True)

aov.to_csv(OUTDIR/f"Fig6_Res34_ANOVA_{cell}_{grp}.csv", index=False)

# Tukey HSD

tuk = pg.pairwise_tukey(dv="value", between="condition", data=sub)

tuk.to_csv(OUTDIR[f"Fig6_Res34_Tukey_{cell}_{grp}.csv", index=False)

# ----------------------------

# Plotting (Figures 5 & 6 bar charts; optional)

# ----------------------------

def barplot_exp1(long_df: pd.DataFrame):

"""For each cell type and condition (control, E+P), show mean±SD across groups."""

df1 = long_df[long_df["experiment"]=="exp1"]

if df1.empty:

return

for cell in sorted(df1["cell_type"].unique()):

for cond in sorted(df1["condition"].unique()):

sub = df1[(df1.cell_type==cell) & (df1.condition==cond)]

if sub.empty:

continue

order = ["siGFP","siERβ","siCOX2","siDual"]

means = [sub[sub.group==g]["value"].mean() for g in order]

sds = [sub[sub.group==g]["value"].std(ddof=1) for g in order]

x = np.arange(len(order))

fig, ax = plt.subplots(figsize=(6.5,4))

ax.bar(x, means, yerr=sds, capsize=4, edgecolor="black")

ax.set_xticks(x); ax.set_xticklabels(order)

ax.set_ylabel("A450 (mean ± SD)"); ax.set_title(f"Figure 5 – {cell}, {cond}")

plt.tight_layout(); plt.savefig(OUTDIR/f"Fig5_{cell}_{cond}_bar.png", dpi=300); plt.close()

def barplot_exp2(long_df: pd.DataFrame):

"""For each cell type and group (siGFP or siDual), show mean±SD across conditions."""

df2 = long_df[long_df["experiment"]=="exp2"]

if df2.empty:

return

for cell in sorted(df2["cell_type"].unique()):

for grp in ["siGFP","siDual"]:

sub = df2[(df2.cell_type==cell) & (df2.group==grp)]

if sub.empty:

continue

cond_order = ["control","E","P","E+P"]

means = [sub[sub.condition==c]["value"].mean() for c in cond_order]

sds = [sub[sub.condition==c]["value"].std(ddof=1) for c in cond_order]

x = np.arange(len(cond_order))

fig, ax = plt.subplots(figsize=(6.5,4))

ax.bar(x, means, yerr=sds, capsize=4, edgecolor="black")

ax.set_xticks(x); ax.set_xticklabels(cond_order)

ax.set_ylabel("A450 (mean ± SD)"); ax.set_title(f"Figure 6 – {cell}, {grp}")

plt.tight_layout(); plt.savefig(OUTDIR/f"Fig6_{cell}_{grp}_bar.png", dpi=300); plt.close()

# ----------------------------

# Run

# ----------------------------

if __name__ == "__main__":

# Stats

fig5_tbl = run_exp1_stats(long)

run_exp2_anova_tukey(long)

# Optional plots

barplot_exp1(long)

barplot_exp2(long)

# Also save the long-format data used

long.to_csv(OUTDIR/"long_triplicates_used_for_stats.csv", index=False)

print("[DONE] Outputs written to:", OUTDIR.resolve())
