# Supplementary material for: Identification of Adeno-Associate Virus (AAV) Serotype for Endometriosis Therapy and Effect of AAV-Mediated RNAi Delivery on Gene Expression and Cell Proliferation in In Vitro Endometrial Cell Culture
Source: Microorganisms. 2025 Sep 13;13(9):2144. doi: 10.3390/microorganisms13092144 (PMC12472493; doi:10.3390/microorganisms13092144)
Supplement: Supplementary file 1 [file microorganisms-13-02144-s001.zip › Supplementary Tables.docx]

**Supplementary Table S1**. ESR2-targeting siRNA sequences screened in ectopic endometrial cells.

| **No.** | **Target gene** | | **siRNA ID #** | **Sense Sequence** | **Antisense Sequence** | **Mol. Wt. (g/mol)** |
| --- | --- | --- | --- | --- | --- | --- |
| 1 | ESR2 | J-003402-13 | 145909 | CGGCUCCAUAUACAUACCUtt | AGGUAUGUAUAUGGAGCCGtg | 13,325.40 |
| 2 | (estrogen receptor beta) | J-003402-14 | 145910 | GCAAGAUCGCUAGAACACAtt | UGUGUUCUAGCGAUCUUGCtt | 13,300.40 |
| **3** |  | **J-003402-15** | **145911** | **CCUUACCUGUAAACAGAGAtt** | **UCUCUGUUUACAGGUAAGGtg** | **13,310.40** |

Three premade siRNAs (IDs 145909, 145910, 145911; VectorBuilder) were tested in ectopic endometrial cells to identify the most effective ESR2-targeting sequence. siRNA 145911 showed the greatest downregulation efficiency and was selected for subsequent experiments

**Supplementary Table S2**. Cohen’s d effect size estimates for proliferation assays; Table S3: qRT-PCR quantification of EGFP expression

| **Cell type** | **Condition** | **Comparison** | **Cohen's d** | **Interpretation** |
| --- | --- | --- | --- | --- |
| Eutopic | Con | siGFP vs siERβ | 16.25 | huge |
| Eutopic | E 1nM | siGFP vs siERβ | 21.599 | huge |
| Eutopic | P 10nM | siGFP vs siERβ | 0 | trivial |
| Eutopic | E 1nM+ P10nM | siGFP vs siERβ | 15.208 | huge |
| Eutopic | Con | siGFP vs siCOX2 | 7.65 | huge |
| Eutopic | E 1nM | siGFP vs siCOX2 | 8.449 | huge |
| Eutopic | P 10nM | siGFP vs siCOX2 | 6.269 | huge |
| Eutopic | E 1nM+ P10nM | siGFP vs siCOX2 | 9.713 | huge |
| Eutopic | Con | siGFP vs siDual | 39.567 | huge |
| Eutopic | E 1nM | siGFP vs siDual | 53.775 | huge |
| Eutopic | P 10nM | siGFP vs siDual | 23.025 | huge |
| Eutopic | E 1nM+ P10nM | siGFP vs siDual | 35.241 | huge |
| Ectopic | Con | siGFP vs siERβ | 12.66 | huge |
| Ectopic | E 1nM | siGFP vs siERβ | 12.551 | huge |
| Ectopic | P 10nM | siGFP vs siERβ | 52.462 | huge |
| Ectopic | E 1nM+ P10nM | siGFP vs siERβ | 7.006 | huge |
| Ectopic | Con | siGFP vs siCOX2 | 15.256 | huge |
| Ectopic | E 1nM | siGFP vs siCOX2 | 13.988 | huge |
| Ectopic | P 10nM | siGFP vs siCOX2 | 54.089 | huge |
| Ectopic | E 1nM+ P10nM | siGFP vs siCOX2 | 6.397 | huge |
| Ectopic | Con | siGFP vs siDual | 19.318 | huge |
| Ectopic | E 1nM | siGFP vs siDual | 17.437 | huge |
| Ectopic | P 10nM | siGFP vs siDual | 23.758 | huge |
| Ectopic | E 1nM+ P10nM | siGFP vs siDual | 7.589 | huge |

**Supplementary Table S3**. qRT-PCR quantification of EGFP expression across AAV serotypes

| **Serotype** | **Mean** | **SD** |
| --- | --- | --- |
| **Eutopic** | | |
| Control 1(cell only) | 104 | 35 |
| Control 2 (reagent only) | 11 | 3 |
| scAAVrh43 | 2061 | 446 |
| scAAVrh39 | 2641 | 801 |
| scAAVrh10 | 683 | 516 |
| scAAVrh | 172 | 117 |
| scAAV9 | 858 | 535 |
| scAAV8 | 973 | 747 |
| scAAV7 | 1098 | 673 |
| scAAV6.2 | 362103 | 26676 |
| scAAV6 | 75919 | 2808 |
| scAAV5 | 8850 | 1150 |
| scAAV4 | 571 | 470 |
| scAAV3 | 31274 | 2203 |
| scAAV2 | 898840 | 17419 |
| scAAV1 | 396 | 317 |
| **Ectopic** | | |
| Control 1(cell only) | 103 | 28 |
| Control 2 (reagent only) | 61 | 16 |
| scAAVrh43 | 2527 | 421 |
| scAAVrh39 | 6227 | 1329 |
| scAAVrh10 | 4032 | 3462 |
| scAAVrh | 1084 | 693 |
| scAAV9 | 1847 | 1191 |
| scAAV8 | 2072 | 1437 |
| scAAV7 | 3150 | 2018 |
| scAAV6.2 | 145495 | 8693 |
| scAAV6 | 58067 | 1285 |
| scAAV5 | 35534 | 6113 |
| scAAV4 | 3524 | 1783 |
| scAAV3 | 25853 | 1000 |
| scAAV2 | 17994481 | 829852 |
| scAAV1 | 1280 | 692 |
| **hGL5 cell** | | |
| Control 1(cell only) | 102 | 23 |
| Control 2 (reagent only) | 257 | 46 |
| scAAVrh43 | 39352 | 6454 |
| scAAVrh39 | 15299 | 1625 |
| scAAVrh10 | 7261 | 4209 |
| scAAVrh | 7241 | 4604 |
| scAAV9 | 11560 | 6033 |
| scAAV8 | 7343 | 3725 |
| scAAV7 | 21122 | 1935 |
| scAAV6 | 147169 | 3432 |
| scAAV4 | 36199 | 4390 |
| scAAV3 | 50011 | 8118 |
| scAAV2 | 3285433 | 53923 |
| scAAV1 | 19658 | 701 |
